# Relational peer victimization and depression symptoms in young adults: longitudinal evidence from before and during the COVID-19 pandemic

**DOI:** 10.3389/frcha.2024.1411304

**Published:** 2024-07-17

**Authors:** Tracy Vaillancourt, Heather Brittain

**Affiliations:** ^1^ Counselling Psychology, Faculty of Education, University of Ottawa, Ottawa, ON, Canada; ^2^ School of Psychology, Faculty of Social Sciences, University of Ottawa, Ottawa, ON, Canada

**Keywords:** relational peer victimization, depression symptoms, COVID-19, longitudinal, young adults

## Abstract

**Introduction:**

Some targets of relational peer victimization become depressed because of their poor treatment. These associations are well documented in youth but are rarely studied in adults.

**Methods:**

The longitudinal pathways between relational peer victimization (being excluded, stonewalled, etc.) and symptoms of depression were examined in a sample of 392 young adults from Ontario, Canada using annual assessments from age 19 to 24. The role of the COVID-19 pandemic was also examined.

**Results:**

Latent curve models with structured residuals indicated that individuals who reported greater relational peer victimization than others also reported more symptoms of depression (between-person association) and those who were more relationally victimized than their expected level were more depressed than expected (within-person association). During the COVID-19 pandemic, the within-time association between relational peer victimization and depression symptoms was reduced. Specifically, accounting for between-person effects and prior individual differences, we found a predicted decoupling of relational peer victimization and depression symptoms in the first year of the pandemic when social non-pharmaceutical interventions (NPIs) were heavily implemented in Ontario, but not the second year, when NPIs were relaxed (but not abandoned).

**Discussion:**

Our findings indicate that the social NPIs implemented in the initial year of the pandemic may have inadvertently led to a positive impact on the association between relational peer victimization and depression symptoms. This finding underscores the importance of minimizing interactions with abusive peers whenever feasible as a strategy to enhance mental well-being.

## 1 Introduction

Peer victimization is a common experience in childhood and adolescence with approximately 30% of youth worldwide reporting being harmed by their peers (1). Peer victimization is also widespread in adulthood with 19.4% of adults reporting being abused (2). One notable difference between peer victimization in childhood vs. adulthood is the form the abuse takes [see (3) for a review]. In childhood, peer victimization tends to be more direct in nature, involving physical and verbal aggression. As children mature, direct forms of aggression are replaced with indirect (i.e., relational) aggression (4), which peaks in adolescence (5), but persists in use across the life span (6), even into old age (7). In the present study, we focused on relational peer victimization which entails behaviour intended to hurt another without direct confrontation. Examples include being socially excluded, being the target of gossip or rumours, or being given the silent treatment.

Relational aggression is commonly used by adolescents and adults because, unlike physical and verbal aggression, which are heavily rebuked by society, relational aggression is tolerated (8, 9). This acceptance stems from the misperception that relational peer victimization is less harmful to targets than physical and/or verbal peer abuse. Even though the use of relational aggression against peers is abided, it is far from benign. Indeed, individuals who are relationally victimized by their peers suffer a host of psychosocial problems such as increased loneliness (10) and anxiety (11, 12), disordered eating (13), somatic complaints (14), and suicidality (15–17). Relational peer victimization also uniquely affects mental health outcomes. For example, Lundh et al. (18) found that targets of relational, but not direct peer victimization, were more likely to experience emotional symptoms over time, controlling for prior symptoms. Researchers have consistently documented that relational peer victimization takes a particular toll on mood, with depression being one of the most common correlates (19, 20) and outcomes in adolescents (21, 22) and correlates in adults (23–25). In fact, meta-analytic findings demonstrate that relational peer victimization is more strongly linked to internalizing problems than direct forms of peer abuse (19). This association is not surprising given the established appreciation that “interpersonal relationships matter for growth and adjustment”, and notably, mood [(26), p. 244].

One of the most common pathways to depression is through interpersonal trauma, which includes peer victimization (27). According to the interpersonal theory of depression, relationship disturbances are an important component in explaining the development of depression because they interact with relationship appraisals to increase stress and conflict in relationships (26). Specifically, experiencing chronic interpersonal dysfunction like relational peer victimization, can lead to internalizing negative feedback from others where individuals become sensitized (i.e., heightened emotions that are more difficult to regulate) to difficult social situations that confer risk for depression (28). Although there is ample support for this model in children and adolescents (19, 20, 27), in adults, little is known about the concurrent and prospective links between relational peer victimization and depression. This paucity in knowledge is curious given that as individuals age, relational aggression becomes the most common form of interpersonal aggression used against peers (6, 8). In the only longitudinal study to date to examine relational peer victimization and depression symptoms, Leadbeater et al. (29) found positive concurrent associations between relational peer victimization and depression symptoms across all five time points assessed (from early adolescence to young adulthood), as well as prospective associations from relational peer victimization to later depression symptoms. In this study, depression symptoms were modeled as a latent growth curve with relational peer victimization as a time-varying covariate. The development of peer victimization was not examined vis-à-vis the development of depression symptoms. In the present study, we examined the concurrent and longitudinal associations between relational peer victimization and depression symptoms in young adults assessed yearly on six occasions using a latent curve model with structured residuals [LCM-SR; (30, 31)]. This analytic approach disaggregates within- and between-person relations (30–33), thus avoiding blending effects into a single estimate (34), which inhibits the interpretation of true individual change.

Depression is the leading cause of disability in adults worldwide (35), affecting 8% of adults and contributing to significant loss of income and difficulty with work, home, and social activities (36). Reducing rates of depression is a global priority (37, 38), which requires knowledge about the conditions of risk. During the pandemic, depression symptoms increased for children and adolescents (39), as well as for adults (40), especially young adults (41) worldwide. Yet, at the same time, there was a global reduction in peer victimization rates in children and adolescents (42), which corresponded with better mental health among targets of bullying (43). Vaillancourt et al. (42, 44) have argued that declines in peer victimization were likely due to the implementation of non-pharmaceutical interventions (NPI) aimed at reducing the spread of SARS-CoV-2. Specifically, in the first year of the pandemic, many countries experienced social lockdown periods which varied in terms of duration and restrictions. In Ontario, Canada, where the present study was conducted, the provincial government implemented some of the longest and most comprehensive social NPIs in the world, beginning in March 2020 and continuing until March 2022 (45). Social NPIs included stay-at-home orders, restrictions on social gatherings and public events, physical distancing, implementation of online learning for elementary, secondary, college, and university students, restrictions on travel, and remote work for non-essential employees, among others.

Although generally examined in relation to their efficacy in reducing virus transmission rates, in the present study, we consider another feature of NPIs. Specifically, we examined the impact of population-level social NPIs on exposure to peer victimization. Social restrictions during the pandemic reduced face-to-face contact with peers, including abusive ones (44). Accordingly, we predicted that rates of relational peer victimization would decline across time as per previous studies (6), but especially during the first year of the pandemic in 2020 when the socialization prospects of young adults were dramatically reduced because NPIs were heavily enforced (45). For example, in the Fall of 2020, only 4% of university students learned in person, a rate which increased to 52% in the Fall of 2021 and to 83% in the Fall of 2022 (46). As another example, bars were closed in March 2020, but by February 2021, were opened with a few restrictions (45).

To assess our hypothesis, we examined relational peer victimization and depression symptoms four years before the pandemic and during the first two years of the pandemic. Depression symptoms were expected to remain stable over time, even though we predicted decreases in peer abuse. This prediction was based on evidence showing that depression increased in adults during the pandemic (40, 41). Relational peer victimization was expected to be concurrently related to depression symptoms at all times assessed before the pandemic, but reduced during the pandemic, based on results from Farrell et al. (43) showing a decoupling of peer victimization and mental health problems in youth during the pandemic. The de-coupling of relational peer victimization and depression symptoms was expected to be present in the first year of the pandemic and not the second year based on changes in socialization opportunities associated with NPI requirement changes. Specifically, by year two of the pandemic, social NPI were less restrictive than in year one (45). Regarding temporal precedence, we expected that relational peer victimization, a notable interpersonal stressor, would predict increases in depression symptoms across time (even during the pandemic), consistent with Leadbeater et al. (29).

## 2 Materials and methods

### 2.1 Participants

Participants were drawn from an on-going Canadian longitudinal study called The McMaster Teen Study, which was designed to examine the links between bullying, mental health, and academic achievement. The original cohort was recruited from a randomized sample of 875 students in Grade 5 (*M*_age_ = 10.91 years; *SD* = 0.36) of which 704 participated in at least one follow-up assessment. Annual assessments are still on-going. For this study, we used data from six waves—age 19 to age 24, which were collected yearly starting in 2016. Age 23 and 24 data collection intersected with the COVID-19 pandemic. The analytic sample was restricted to participants who provided data on relational peer victimization and symptoms of depression (at one or more time points from age 19 to age 22), as well as one or both time points during the pandemic. Eight participants were flagged for invalid responses and their data were omitted from the respective time point. The final analytic sample was comprised of 392 primarily White [78.1%; 15.3% non-White including Middle Eastern Canadian, African/West-Indian Canadian (Black), Asian-Canadian, South-Asian-Canadian, Native-Canadian, South/Latin American Canadian, or Other; 6.6% missing] middle-class^1^ participants (61.2% women). Participants could opt out of participation at any given year yet remain enrolled and take part in future time points. Of the analytic sample, 62.5% (*n *= 245) had complete data across the six time points (i.e., relational peer victimization and depression scores within each wave; missing both relational peer victimization and depression scores within each wave: 9.4% at age 19, 8.2% at age 20, 8.4% at age 21, 7.9% at age 22, 8.7% at age 23, 6.1% at age 24). The overall rate of missing data across the six waves was 11%. Most participants in the analytic sample (94.5%) lived in Ontario during the first year of the pandemic (2020) and 93% lived in Ontario during the second year of the pandemic (2021).

### 2.2 Procedure

Ethics approval was received from the relevant university ethics boards every year, and initial ethics approval was received from the relevant school board. Participants were recruited from 51 randomly selected schools from a single school board. From Time 1 (age 10) to Time 8 (age 18) parents provided annual consent for themselves, and their children and participants provided assent. From Time 9 (age 19) on, participants provided consent. Participants completed annual surveys (requiring approximately 30–45 min to complete) either by paper or online beginning at Time 2 (age 11). Compensation in the form of a gift card (or e-transfer in later years) increased incrementally (e.g., Time 2: $10; Time 14: $75).

### 2.3 Measures

#### 2.3.1 Relational peer victimization

Relational peer victimization was measured using the valid and reliable 35-item Indirect Aggression Scale Target Version (47). Participants were asked about how often they experienced relational peer victimization in the context of adult social interactions and close interpersonal relationships. Sample items included “Purposefully left me out of activities” and “Talked about me behind my back”. Each item was rated on a five-point scale (1 = *never* to 5 = *always*) and averaged to create a composite with higher scores indicating higher relational peer victimization. The Cronbach's Alpha (α) for age 19 to age 23 was .97 and at age 24 was .98. The McDonald's Omega (ω) was identical at each time point.

#### 2.3.2 Depression symptoms

Self-reported depression symptoms were measured using the depression subscale of the Self-Report of Personality - College Version [SRP-COL; (48)] of the Behavioural Assessment System for Children-2 (BASC-2). Items were rated as *true *= 2 or *false *= 0 for 9 items (e.g., “Nothing is fun anymore”) and on a 4-point Likert-type scale of *never* = 0, *sometimes* = 1, *often* = 2 and *almost always* = 3 for 4 items (e.g., “I feel sad”). We created a composite score by summing the 13 items with higher scores reflecting more symptoms of depression. Cronbach's *α* for the depression items were very good at each time point (age 19 *α* = .92, age 20 *α* = .90, age 21 *α* = .91, age 22 *α* = .90, age 23 *α* = .91, and age 24 *α* = .89). The McDonald's Omega ω were identical at each time point.

#### 2.3.3 Covariates

We accounted for child maltreatment (physical and sexual) in our analyses so that associations with depression could be attributed to relational peer victimization and not another common form of interpersonal trauma that has been shown to be independently associated with increased peer victimization (49) and depression (50) in adults.

Specifically, the Childhood Experiences of Violence Questionnaire - Short Form [CEVQ-SF; (51)] was used to assess exposure to child maltreatment. This retrospective measure consists of 7 items on physical and sexual abuse answered along a 5-point scale (*never* to *more than 10 times*). A minimum score of 3–5 times for one or more items was used to classify abuse, except for sexual abuse, which required any experience to qualify as abuse (51, 52). The CEVQ-SF was administered at age 19 and again at age 20 for participants who did not report on this measure the previous year. Participants were categorized into two groups using established cut off criteria (51, 52), where 1 = *any physical or sexual maltreatment or both* and 0 = *no physical or sexual maltreatment*.

Gender was also accounted for in our analyses (women = 1 and men = 0) because women are more likely than men to be targets of relational peer victimization (8) and to be depressed (36). We also accounted for local weekly COVID-19 case counts from the community in which each participants resided, following Krygsman et al. (53).

### 2.4 Analytic plan

Analyses were performed in Mplus Version 8.0 (54) using full information maximum likelihood estimation (FIML; maximum likelihood estimation with missing data). The following fit indices were considered when evaluating each model: the comparative fix index (CFI), the Tucker–Lewis Index (TLI), the root mean square error of approximation (RMSEA), the standardized root mean square residual (SRMR), the χ^2^ test of significance, and the Akaike information criterion (AIC). CFI and TLI values >0.95 indicate adequate model fit, RMSEA values <0.06 indicate close fit, SRMR values <0.08 indicate good fit, and lower AIC values indicate a better fitting model (55, 56). Differences between nested models were assessed with the chi-square difference test. Parameter constraints within models were examined with the Wald test.

Models were organized as LCM-SR (31) to investigate within-person associations among relational peer victimization and symptoms of depression within and across time while also accounting for between-person associations. Models were estimated with growth parameters, representing individual starting values and change over time, and structured residuals of each observed variable (i.e., latent variables with a single observed item), representing individual time-dependent deviations from their own trajectories. We first examined univariate models of relational peer victimization and symptoms of depression independently by determining the best fit to model growth (i.e., intercept only, linear, and quadratic) with the addition of autoregressive paths between structured residuals, testing if constant or free parameter estimates were a better fit. We then combined the best-fitting univariate curves into a multivariate model with the addition of covariances between all growth factors (between-person associations) and within-time between the structured residuals of each variable (e.g., age 20 relational peer victimization with age 20 depression symptoms; within-person associations), setting age 20 to age 24 residual covariances equal. We examined if freeing residual covariances resulted in better fit, then tested the addition of cross-lagged paths (constant and free) between structured residuals (e.g., age 21 relational peer victimization to age 22 depression symptoms). Where parameter estimates were found to be different (i.e., freeing estimates resulted in better fit), we examined differences using the Wald χ^2^ test. Using the best fitting model, a final conditional model was estimated by regressing growth factors on the correlated time-invariant covariates of gender and child maltreatment. Age 22 and 23 relational peer victimization and depression symptoms structured residuals were regressed on the (natural log transformed) COVID-19 case counts for the respective year.

## 3 Results

### 3.1 Missing data analysis

The analytic sample (*N* = 392) was compared to participants who were in the longitudinal sample but not included in the present study (i.e., no data in T9–T14) on demographic variables and study variables at the relevant time points as well as baseline depression symptoms. Chi-square tests were used to examine gender differences and parental education and *t*-tests (two-sided) were used to examine household income and depression and relational peer victimization. There was a significant difference by gender, χ^2^(1) = 24.511, *p *< .002, *Phi* = .186, such that the analytic sample was composed of a higher proportion of women (61.2%) than men (38.8%) and the non-analytic sample was composed of a higher proportion of men (57.5%) than women (42.5%). Compared to participants who were not selected in the current study, the analytic sample reported significantly higher household income *t*(572.584) = −4.107, *p* < .001, *d *= −0.329 (*M* = 5.724 for nonanalytic sample and *M* = 6.478 for analytic sample) and parental education, χ^2^(679,4) = 38.143, *p* = .001. The nonanalytic sample had a higher proportion of parents reporting high school education (27.2%) or less (7.9%) than the analytic sample (17.2% and 1.9%, respectively) whereas the analytic sample had a higher proportion of parents reporting university undergraduate (29.7%) or graduate degrees (12.5%) than the non-analytic sample (16.2% and 7.6%, respectively). The analytic sample and nonanalytic sample reported similar Time 1 depression scores, *t*(647) = 1.046, *p* = .296, *d *= 0.083. There were no differences found on depression scores, *p*s = .064–.881, *d*s* *= −.035–.382, or relational peer victimization scores, *p*s = .255–.936, *d*s* *= .018–.273, reported by those in the analytic sample and those not included in the analytic sample.

Within the analytic sample, cases were evaluated on key variables of interest (i.e., depression, relational peer victimization, gender, child maltreatment). Little's MCAR test indicated that missing data were not missing completely at random, χ^2^(360) = 452.709, *p* = .001. We further examined if missingness on each variable was related to observed scores on other variables. Missing depression scores at ages 19, 20, 21, and 22 were associated with subsequently higher observed relational peer victimization scores at one or more time points compared to those not missing. Missing depression scores at ages 21 and 22 were associated with higher observed depression scores the following year. Higher relational peer victimization scores at age 21 were associated with age 22 missing depression scores. Missing relational peer victimization scores at ages 19 and 20 were associated with higher scores on observed relational peer victimization scores at one or more subsequent time points and missing age 21 relational peer victimization were related to higher depression scores the following year. Those missing age 24 depression or relational peer victimization scores were more likely to have lower prior depression scores than those with data at age 24. Men and women did not differ on the average number of missing scores, *t*(293.118) = 1.887, *p* = .060, *d* = .201 (*M* = 1.612 for men and *M* = 1.158 for women). Individuals reporting experiencing child maltreatment had a similar average number of missing survey items than those who did not, *t*(98.225) = −1.827, *p* = .071, *d* = -.265 (*M* = 1.507 for those with a history of child maltreatment and *M* = 0.993 for those without). We assumed data were missing at random. All variables that were associated with missingness were included as auxiliary variables in models that did not already contain the variables (i.e., depression scores in the univariate relational peer victimization models and peer victimization in the univariate depression symptoms models), satisfying conditions of missing at random.

### 3.2 Descriptive statistics

Descriptive statistics and correlations between observed variables are found in Table 1. All correlations within and across constructs were statistically significant, *p *< .001. In each year of the study, with the exception of age 22, *t*(355) = −1.929, *p *= .054, -*d *= .209, women experienced higher symptoms of depression than men, *p*s < .006 (*d *= −.359 to −.288). They also experienced higher levels of relational peer victimization, *p*s < .024 (*d *= −.458 to −.247). Mean levels of depression symptoms did not differ between any time points. Relational aggression scores tended to decrease with time; age 19 scores were highest, followed by age 20 (which did not differ from age 21), followed by ages 22, 23, and 24 (which did not differ). Across all time points, individuals with a history of child maltreatment reported higher symptoms of depression, *p*s < = .001 (*d *= −.704 to −.584) and higher levels of relational peer victimization, *p*s < .001 (*d *= −.928 to −.627) than those who did not report experiencing any form of child maltreatment. The proportion of those who experienced child maltreatment did not vary by gender, χ^2^(1) = 1.579, *p *= .209.

**Table 1:** Descriptive statistics and correlations.

	DEP age 19	DEP age 20	DEP age 21	DEP age 22	DEP age 23	DEP age 24	VIC age 19	VIC age 20	VIC age 21	VIC age 22	VIC age 23	VIC age 24	Min.	Max.	*M*	*SD*	%
DEP age 19	1												0.00	30.00	5.565	6.314	
DEP age 20	.684	1											0.00	30.00	5.555	5.897	
DEP age 21	.591	.681	1										0.00	29.00	5.756	6.198	
DEP age 22	.561	.596	.697	1									0.00	29.00	5.560	5.810	
DEP age 23	.592	.697	.705	.754	1								0.00	29.00	5.528	5.923	
DEP age 24	.536	.605	.611	.642	.754	1							0.00	30.00	5.833	5.821	
VIC age 19	.535	.444	.383	.385	.446	.402	1						1.00	5.00	1.673	0.614	
VIC age 20	.395	.470	.347	.350	.405	.414	.707	1					1.00	5.00	1.607	0.596	
VIC age 21	.372	.452	.489	.449	.443	.450	.655	.713	1				1.00	5.00	1.601	0.599	
VIC age 22	.253	.379	.386	.491	.457	.401	.590	.637	.732	1			1.00	5.00	1.552	0.566	
VIC age 23	.312	.335	.414	.438	.452	.418	.571	.560	.667	.733	1		1.00	5.00	1.541	0.605	
VIC age 24	.307	.370	.407	.413	.471	.528	.560	.563	.655	.676	.714	1	1.00	5.00	1.525	0.597	
Gender^a^																	38.8% men; 61.2% women
Maltreatment^b^																	77.8% no; 22.2% yes

DEP, depression; VIC, relational peer victimization; Min., minimum; Max., maximum.

^a^ 0 = men, 1 = women.

^b^ 0 = did not report having experienced child maltreatment, 1 = reported having experienced child maltreatment. All correlations are statistically significant at *p *< .001.

### 3.3 Univariate unconditional curves

Univariate quadratic growth in depression fit the data best compared to linear and intercept only models (see Table 2 for model fit and model comparison statistics). The average trajectory was characterized as stable over time (significant intercept mean, non-significant slope and quadratic mean) with significant variation in all growth factors (i.e., intercept, slope, quadratic). The addition of constant autoregressive paths between structured residuals resulted in an improvement in model fit. In this model, the covariances between growth parameters and variances of linear slope and quadratic factors were no longer statistically significant. Freeing the autoregressive paths resulted in a not positive definite latent variable covariance matrix. To aid in model estimation we fixed the (non-significant) variance of the quadratic factor to 0, resulting in a model that did not significantly differ from the quadratic growth model with constant autoregressive paths. Freeing the autoregressive paths in this model did not result in model improvement. The quadratic (fixed variance) growth model with constant autoregressive paths was selected as the final univariate depression model.

**Table 2:** Summary of model Fit statistics for the univariate latent curve models.

Model	χ^2^	*df*	*p*	CFI	TLI	RMSEA (90% CI)	SRMR	AIC	Comp.	Δχ^2^	Δ*df*	*p*
Depression symptoms												
1. Intercept	76.958	19	<.001	0.957	0.966	0.088 (.068, .109)	0.051	12,445.578	–	** **	** **	** **
2. Linear growth	40.295	16	<.001	0.982	0.983	0.062 (.039, .087)	0.040	12,414.914	2 vs. 1	36.663	3	<.001
3. Quadratic growth	19.780	12	.071	0.994	0.993	0.041 (.000, .072)	0.025	12,402.400	3 vs. 2	20.515	4	<.001
4. Constant autoregressive paths	13.195	11	.281	0.998	0.998	0.023 (.000, .060)	0.024	12,397.814	4 vs. 3	6.585	1	.010
5. Revised constant autoregressive paths^a^	16.604	14	.278	0.998	0.998	0.022 (.000, .056)	0.029	12,395.224	4 vs. 5	3.409	3	.333
6. Free autoregressive paths	15.214	10	.125	0.996	0.994	0.036 (.000, .071)	0.027	12,401.833	6 vs. 5	1.390	4	.846
Relational peer victimization												
1. Intercept	133.773	19	<.001	0.918	0.935	0.124 (.015, .144)	0.076	2,617.111	–			
2. Linear growth	35.522	16	.003	0.986	0.987	0.053 (.031, .081)	0.042	2,524.859	2 vs. 1	98.251	3	<.001
3. Quadratic growth	14.807	12	.252	0.998	0.997	0.024 (.000, .060)	0.017	2,512.144	3 vs. 2	20.715	4	<.001
4. Constant autoregressive paths^b^	7.038	11	.796	1.000	1.004	0.000 (.000, .035)	0.016	2,506.375	4 vs. 3	7.769	1	.005
5. Free autoregressive paths	5.242	7	.631	1.000	1.003	0.000 (.000, .052)	0.017	2,512.579	5 vs. 4	1.796	4	.773

χ^2^, Chi-square; CFI, comparative fit index; TLI, Tucker-Lewis index; RMSEA, root mean square error of approximation; SRMR, standardized root mean square residual; Comp., model comparison; Δ*df*, difference in degrees of freedom.

^a^ Final univariate model depression symptoms.

^b^ Final univariate model relational peer victimization.

Like the trajectory of depression symptoms, the univariate curve for victimization was also best modeled by quadratic growth with the model yielding significant means for the intercept and slope (declining) and significant variances for all growth parameters. The addition of constant autoregressive paths between structured residuals improved the model. In this model, the variance of the linear slope and quadratic factors were non-significant. Subsequently freeing the paths did not result in a change in model fit. The quadratic growth model with constant autoregressive paths was selected as the final univariate curve.

### 3.4 Bivariate LCM-SR of victimization and depression symptoms

Combining the final univariate models into a bivariate model, we included covariance terms between intercept and growth factors of victimization and depression symptoms and added covariances between within-time residuals, with covariances for age 20 to age 24 held constant. The model resulted in a non-positive definite latent variable covariance matrix due to a non-significant negative variance for the peer victimization quadratic factor. To aid in model estimation, the variance of the quadratic factor was constrained to 0 (see Table 3 for model fit and model comparison statistics). With this constraint in place, the base model had excellent fit to the data χ^2^(58) = 72.011, *p *= .102, CFI = .995, TLI = .995, RMSEA = .025 90% CI = (.000,.042), SRMR = .026, AIC = 14,598.855. Freeing the constrained residual covariances resulted in an improvement in model fit, χ^2^(54) = 61.807, *p *= .217, CFI = .997, TLI = .997, RMSEA = .019 90% CI = (.000,.039), SRMR = .025, AIC = 14,596.651, Δχ^2^(4) = 10.204, *p *= .037. The addition of constant cross-lagged paths did not result in a change in model fit^2^, Δχ^2^(2) = 4.343, *p *= .114, nor did freeing these parameter estimates, Δχ^2^(8) = 10.129, *p *= .256.

**Table 3:** Summary of model Fit statistics for the bivariate latent curve models.

Model	χ^2^	*df*	*p*	CFI	TLI	RMSEA (90% CI)	SRMR	AIC	Comp.	Δχ^2^	Δ*df*	*p*
1. Covariance (constant residual covariance)	72.011	58	.102	0.995	0.995	0.025 (.000, .042)	0.026	14,598.855	–			
2. Covariance (residual covariance free)^a^	61.807	54	.217	0.997	0.997	0.019 (.000, .039)	0.025	14,596.651	2 vs. 1	10.204	4	.037
3. Cross lagged paths (constant)	57.464	52	.280	0.998	0.998	0.016 (.000, .037)	0.024	14,596.308	3 vs. 2	4.343	2	.114
4. Cross lagged paths (free)	47.335	44	.338	0.999	0.998	0.014 (.000, .037)	0.023	14,602.179	4 vs. 3	10.129	8	.256
5. Conditional model^b^	117.28	94	.052	0.993	0.991	0.025 (.000, .039)	0.042	17,358.031	–			

χ^2^, Chi-square; CFI, comparative fit index; TLI, Tucker-Lewis index; RMSEA, root mean square error of approximation; SRMR, standardized root mean square residual; Comp., model comparison; Δ*df*, difference in degrees of freedom.

^a^ final unconditional model.

^b^ final conditional model.

The final model was determined to consist of intercept, slope, and quadratic^3^ factors for relational peer victimization and depression symptoms at the between-person level, and constant autoregressions within peer victimization and within depression symptoms as well as time varying within-time covariances between peer victimization and depression symptoms structured residuals at the within-person level. The peer victimization intercept was significantly negatively related to peer victimization slope in standardized terms (cov = −0.007, s.e. = 0.005, *p *= .148; *r *= −.288, s.e.* *= 0.133, *p *= .030); those starting higher than others had more steeply declining slopes than those starting lower. The intercepts of relational peer victimization and depression symptoms were related (cov = 1.365, s.e.* *= 0.209, *p *< .001; *r *= .585, s.e.* *= 0.056, *p < *.001) indicating that individuals starting higher on relational peer victimization than others also reported starting higher on depression symptoms than others. The unstandardized covariance between slopes of relational peer victimization and depression symptoms was marginally significant whereas the standardized correlation was significant (cov = 0.013, s.e. = 0.008, *p *= .113; *r *= .589, s.e. = 0.297, *p *= .047).

At the within-level, autoregressive paths in relational peer victimization were positive and consistent across time (*b = *0.243, s.e. = 0.044, *p < *.001; βs = .243, .264, .261, .215, and .256). Autoregressive paths for depression were also positive and consistent over time (*b *= 0.185, s.e. = 0.042, *p *< .001; βs = .223, .168, .202, .219, and .165). Individuals who scored higher (or lower) than their predicted score of relational peer victimization at one time point were likely to score higher (or lower) than their predicted score at the following, similarly for depression symptoms.

The within-time associations between structured residuals were positive and significant at all pre-pandemic time points (age 19 *cov *= 0.789, s.e. = 0.151, *p *< .001, *r* = .484, s.e. = .067, *p < *.001; age 20 *cov *= 0.290, s.e. = 0.085, *p *= .001, *r* = .227, s.e. = .060, *p < *.001; age 21 *cov *= 0.339, s.e. = 0.086, *p *< .001, *r* = .260, s.e. = .058, *p < *.001; age 22 *cov *= 0.287, s.e. = 0.072, *p *< .001, *r* = .260, s.e. = .059, *p *<* *.001). During the first pandemic year, the association was not statistically significant (age 23 *cov *= 0.029, s.e. = 0.072, *p *= .668, *r* = .027, s.e. = .067, *p *=* .*687) but was during the second year of the pandemic (age 24 *cov *= 0.287, s.e. = 0.088, *p *= .001, *r* = .252, s.e. = .069, *p *<* *.001). Examining the magnitude between residual covariances across time indicated that the age 23 (pandemic year 1) covariance between victimization and depression symptoms was significantly different from pre-pandemic assessments [i.e., age 22, Wald χ^2^(1) = 6.236, *p *= .013; age 21, Wald χ^2^(1) = 7.651, *p *= .006; age 20, Wald χ^2^(1) = 5.424, *p *= .012] as well as from the second year of the pandemic [i.e., age 24, Wald χ^2^(1) = 4.998, *p *= .025]. No other differences were found.

**Figure 1:**
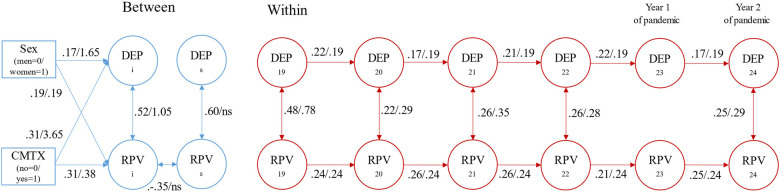
Depression symptoms and relational peer victimization conditional bivariate latent curve model with structured residuals. DEP, depression; RPV, relational peer victimization; CMTX, child maltreatment. Estimates represent standardized/unstandardized values. Only statistically significant paths are depicted tin the figure for ease of visualization. Time invariance constraints include autoregressive paths for depression symptoms and for relational peer victimization. Sex: men = 0 and women = 1. Child Maltreatment: no = 0 and yes = 1.

Finally, we examined the conditional bivariate LCM-SR including time invariant covariates of gender and child maltreatment and time varying covariates of COVID case counts for ages 23 and 24. The model fit the data well, χ^2^(94) = 117.280, *p *= .052, CFI = .993, TLI = .991, RMSEA = .025 90% CI = (.000,.039), SRMR = .042, AIC = 17,358.031. Parameter estimates did not substantively differ between the conditional and unconditional models. In this final model case counts were not related to peer victimization or depression symptoms at either pandemic time point. Gender and child maltreatment were significantly related to the intercepts of relational peer victimization (Gender: *b *= 0.193, s.e. = 0.058, *p *= .001; *β* = .191, s.e. = 057; maltreatment *b *= 0.378, s.e. = 0.074, *p *< .001; *β* = .306, s.e. = .058) and depression symptoms (Gender: *b *= 1.648, s.e. = 0.578, *p *= .004; *β* = .170, s.e. = .059; maltreatment *b *= 3.645, s.e. = 0.723, *p *< .001; *β* = .306, s.e. = .059); being a woman and having experienced child maltreatment were risk factors for elevated scores. The rates of change over time in relational peer victimization and depression symptoms were not related to gender and child maltreatment. Compared to the results of the unconditional model, parameter estimates were similar in magnitude. Results of the conditional model are show in Figure 1.

## 4 Discussion

The link between relational peer victimization and depression symptoms has been studied extensively in children and adolescents [see meta-analysis by (19)], but rarely in adults. Examining these links in adulthood is important because relational aggression is the most common form of interpersonal aggression used in adulthood (6, 8) and because this form of abuse is associated with depression (23–25, 29). One way to prevent the onset of depression is to reduce individuals' exposure to interpersonal violence, including relational aggression. This assertion is based on longitudinal studies demonstrating: (1) a causal link between exposure to interpersonal abuse and the development of mental health difficulties in youth (57); and (2) that interpersonal violence is associated with higher rates of depression than other forms of trauma (58).

In the present study, we examined relational peer victimization and depression symptoms in early adulthood using LCM-SR that permitted the separation of between- and within-person effects. Controlling for gender and prior child maltreatment, established correlates of peer victimization (49) and depression (36, 50), we found that at the between-person level, individuals who were more victimized by their peers also reported higher symptoms of depression than others. This finding replicates other studies showing that relational peer victimization is correlated with depression (23–25, 29). In keeping with the established literature, we found that women experienced higher symptoms of depression and higher levels of relational peer victimization than men (8, 36). Also, individuals with a history of child maltreatment reported higher symptoms of depression (59, 60) and higher levels of peer victimization (61) than those who did not report experiencing any form of child maltreatment. These replicated findings provide support for the validity of our results.

In examining associations between annual assessments of relational peer victimization, at the within-person level, deviations from a person's trajectory were consistently tied to deviations the following year; if someone was lower than expected at one time point, they were also lower than expected the following year, and the strength of the association did not change over time. In the univariate latent trajectory of relational peer victimization, the variance of the growth factor parameter estimates became non-significant with addition of autoregressive paths. The differences in between-person growth may have been partially accounted for by within-person carryover effects. The positive estimate could also “indicate the stability of the rank-order of individual deviations” [(32), p. 105]. At the mean level, we found that relational peer victimization generally declined with age, but contrary to our prediction, the rate of decline during the pandemic was not greater than before. Specifically, relational peer victimization was highest at age 19 and rates were higher at ages 20 and 21 (which did not differ) than at ages 22, 23, and 24 (which did not differ from each other). In other longitudinal studies, peer victimization has been shown to decline with age (62, 63). The global decline could reflect the dissolution of toxic peer relationships. That is, adults who were abused by their peers may have withdrawn from or avoided these relationships, which thereby reduced their exposure to peer abuse over time.

Regarding symptoms of depression, we observed predicted stability over time, including throughout the first two years of the pandemic. We predicted stability because depression is influenced by many factors including the stress of living through a global pandemic (40). This result was present when examining mean levels over time, as well as in the within-person effects resulting from our models. Longitudinal studies on depression point to an unfortunate entrenchment that begins in adolescence and persists until adulthood (64–68). Depression symptoms have been shown to peak in early adulthood, decline in middle adulthood, and then increase in older adulthood (69). This stability and developmental pattern highlight the importance of intervening early. One way to improve mental health is through the avoidance of abusive peers.

We expected and found a decoupling of relational peer victimization and depression symptoms in the first year of the pandemic, but not the second year, accounting for between-person effects and prior individual differences. Of note, during the first year of the pandemic, the within-time association was not statistically significant, and the magnitude of the association was different from all other time points including the second year of the pandemic. That is, deviations from individuals’ own trajectories of relational peer victimization were not associated with deviations from their trajectories of depression symptoms during year one of the pandemic. Moreover, the zero-order correlations between relational peer victimization and depression symptoms were moderate at each time point, which were replicated by a positive correlation in intercepts in our model. Taken together, these findings suggest that social restrictions during the first year of the pandemic may have had an unintended positive effect on the association between mood and relational peer victimization [see also (43)].

Finally, changes in relational peer victimization did not predict increases in future depression symptoms or the reverse. This null finding may be related to our unique analytic approach in which we accounted for between-person effects and trait-like individual differences. It has been shown that if the between-person proportion of variance is small, the cross-lagged paths in the two models will be similar thus providing support for the same conclusions, but if the between-person effects account for a large proportion of the variance, as they did in the present study, there is less variance for the within-person estimates, leading to higher uncertainty and lower power to detect effects. Therefore, different cross-lagged paths may be found, leading to different conclusions about temporal priority (33, 70). At the correlation level, which does not separate the within-person associations from the between-person effects, relational peer victimization was always positively associated with future depression symptoms [interpersonal risk model (victimization → depression); (29)] and the reverse [symptoms-driven model (depression → victimization); (71)], suggesting bi-directional effects (11). Nevertheless, when these features (i.e., between-person effects or prior within-person associations) were attended to, cross-lagged effects were not found. In cases where the trait-like between-person components do not exist, results will be minimally impacted by the additional model parameters; however, failure to account for between-person effects when they do in fact exist can lead to vastly different inferences from models where variability is separated. Moreover, the separation of within- and between-person variability should be considered when examining constructs that demonstrate high-rank order stability like depression (72). These important methodological points notwithstanding, a better design might be to assess relational peer victimization and depression symptoms in closer proximity, as assessing these relations over the course of a few weeks or months could yield different results. It is likely that individuals endure toxic relationships for some time before leaving them. This would likely result in positive cross-lagged associations between relational peer victimization and depression in the short term. Moreover, there is emerging evidence that ambivalent relationships, which consist of a mix of positive and negative features, are in fact more problematic for health outcomes than negative relationships *per se* (73). Perhaps ambivalent relationships should be examined across days or weeks, and not years, to capture their true impact on mental health.

### 4.1 Limitations

Our study has many strengths, including the use of LCM-SR and the examination of the most common form of aggression used against peers in adults—relational aggression. Still, there are limitations that ought to be considered when interpreting our findings. One, we may be underpowered to detect cross-lagged effects as models accounting for between-person variation often require sample sizes of 1,000 participants or more (70). Two, longitudinal studies are vulnerable to attrition that can lead to biased estimates and erroneous conclusions about temporality (74). In our study, dropout was systematic and thus generalizability is challenged. We found that participants in the analytic sample had higher socio-economic indicators (i.e., household income and parental education) than those not included in the study. Thus, it is possible that the associations found in this study would be more pronounced for individuals from lower socioeconomic backgrounds as they are more likely to experience internalizing problems compared to those with higher incomes (75). Three, we did not directly examine the effect of social NPIs on participants but rather extrapolated from what happened at the population level in Ontario. It is possible that other moderating or mediating effects influenced our results. Having a romantic partner, for example, could act as a buffer or as a risk factor for the development and maintenance of depression symptomatology depending on the quality of the relationship (76). Relatedly, peer victimization in another context like the workplace could amplify associations between peer abuse and depression symptoms (77). Accounting for childhood peer experiences is also worthy of consideration given the continuity of victimization across the lifespan. Brendgen et al. (78) found that peer victimization in late childhood and adolescence predicted victimization in college and at work. Four, during the pandemic, social media use increased notably (79) but cyber-victimization rates remained stable (80). The introduction of social NPIs during the pandemic would not have restricted online interactions, including problematic ones, which we did not assess. Finally, our analytic approach precludes an examination of heterogeneity, which should be expected. For example, researchers have shown that young adults with preexisting mental health concerns improved or had similar mental health during the pandemic, whereas individuals without preexisting mental health concerns declined in their mental health (53, 81). To address the limitations identified, future studies should consider the following strategies to improve the robustness and generalizability of our findings: (1) increase sample size, (2) mitigate attrition [see (74)], (3) enhance generalizability by including participants from diverse backgrounds, (4) directly examine NPIs and other moderators, (5) access online social interaction during the pandemic, and (6) consider heterogeneity in analytic approaches (which will require a larger sample size).

### 4.2 Conclusion

The longitudinal pathways between relational peer victimization and symptoms of depression were examined with a particular focus on how pandemic social NPIs at the population level may have influenced within- and across-time associations. Findings revealed a separation between relational peer victimization and depression symptoms during the initial year of the pandemic in Ontario, characterized by stringent implementation of social NPIs. However, this decoupling was not observed in the second year of the pandemic when NPIs were eased, albeit not completely discontinued. This result highlights that one way to improve mental health is to avoid, when possible, abusive peers.

## Data Availability

The raw data supporting the conclusions of this article will be made available by the authors, without undue reservation.

## Footnotes

^1^SES is based on initial sample characteristics (see redacted).

^2^The addition of cross-lagged paths was also examined by adding only one direction of paths at a time (i.e., from victimization to depression symptoms or from depression symptoms to victimization) and comparing to the prior model. All results were also not statistically significant, *p*s < .05.

^3^Variance of the quadratic factors was constrained to zero for model identification.
